# Addressing Challenges in Standardizing Helicobacter pylori Treatment Protocols: Importance and Review

**DOI:** 10.7759/cureus.59394

**Published:** 2024-04-30

**Authors:** Ranjot Kaur, Carlos Leon Guevara

**Affiliations:** 1 Department of Biology, University of the Fraser Valley, Abbotsford, CAN; 2 Health Sciences, Fairleigh Dickinson University, Vancouver, CAN

**Keywords:** bacteria, patient compliance, helicobacter pylori infection treatment, gastric cancer, covid-19 disease, neem, antibiotic resistance, helicobacter pylori

## Abstract

*Helicobacter pylori*, one of the top carcinogens, is associated with most cases of gastric cancer-related deaths worldwide. Over the past two decades, the rising rates of antibiotic resistance in the bacterium have reduced the efficacy of conventional antibiotic-based treatments. This underscores the urgency for continued research and novel treatment approaches. Establishing a worldwide accepted physician guideline for antibiotic prescription is crucial to combat antibiotic resistance and improve *H. pylori *infection management. Therefore, it is important to address the challenges that complicate the establishment of a universally accepted treatment protocol to prescribe an antibiotic regimen to eradicate *H. pylori*. The answers to the questions of why conventional standard triple therapy remains a first-line treatment choice despite its low efficacy, and how different factors affect therapy choice, are needed to identify these challenges. Hence, this review addresses concerns related to *H. pylori* treatment choice, role of antibiotic resistance and patient compliance in treatment outcomes, first-line vs. second-line therapy options, and methods for enhancing existing treatment methods. We also present a chart to aid antibiotic treatment prescription, which may support physician guidelines in this aspect. Eradication of *H. pylori* and patient adherence is paramount in overcoming antibiotic resistance in the bacterium, and our chart summarizes key considerations and suggests novel approaches to achieve this goal.

## Introduction and background

*Helicobacter pylori* is a Gram-negative, microaerophilic bacterium that colonizes the gastric mucosa of about half of the human population [1]. The incidence of *H. pylori* infection varies around the world, inflicting more burden in developing countries where up to 80% of the adult population may be infected [2]. Most of the infected people stay asymptomatic while up to 10% of those affected may experience clinical complications including peptic ulcers which may progress to gastric atrophy, gastric intestinal metaplasia, and gastric cancer [2-4]. Conteduca et al. reported that the risk of gastric cancer in people infected with *H. pylori* is three to six times higher than those uninfected [3]. *H. pylori *infection is associated with 63.4% of the stomach cancer cases worldwide [5] and amounts to 5.5% of the global cancer burden [2]. This makes *H. pylori* a major public health concern. In 2015, at the Kyoto *H. pylori* conference, it was recommended that all *H. pylori* infections should be eradicated unless there are circumstances like comorbidities, significant re-infection rates, and other priorities with higher burdens of disease in a particular population [4,6]. The World Health Organization (WHO) and the International Agency for Research on Cancer classified *H. pylori* as a group 1 carcinogen associated with gastric cancer [2,7]. The eradication of *H. pylori* infections has become increasingly important ever since this classification and the urgency to deal with the pathogen continues to grow, especially in the context of the recent COVID-19 (coronavirus disease) pandemic [8].

The number of *H. pylori*-infected people is still high due to population growth and re-infection associated with poor eradication levels and low socioeconomic status, especially in developing countries [9,10]. Hence, it is essential to devise effective strategies to eradicate *H. pylori*. The human stomach serves as the primary reservoir of *H. pylori *infection [11,12]. *H. pylori* can live in the stomach for decades and are thought to be transmitted by human-to-human contact since the bacterium has a very narrow host range [11,12]. According to Eusebi et al., although some environmental factors may facilitate *H. pylori* transmission, interpersonal transmission remains the main route [13]. *H. pylori* sequencing and genomic studies on isolates collected from infected patients revealed that more transmission occurred between members of the same household as well as their close relatives [13]. Hence, low socioeconomic status in developing countries can be identified as a risk factor for increased transmission of *H. pylori* as living in crowded conditions may facilitate intrafamilial transmission [13,14]. The results of a very recent study by Zandian et al., on *H. pylori*-infected patients in Iran, further supported that poor socioeconomic status and low education levels were important risk factors for increased rates of *H. pylori* infection transmission [15].

The COVID-19 outbreak was a global public health emergency, declared a pandemic by the WHO on March 11, 2020, and has raised further concerns regarding *H. pylori* infections. It has been one of the deadliest pandemics with more than 4.8 million deaths [8]. Most serious forms of COVID-19 infection were diagnosed in patients with underlying medical conditions [8]. Recent studies [8,16] have established the association of *H. pylori* and associated extra gastric disorders with the clinical symptoms of COVID-19 disease. Gonzalez et al. highlighted that existing *H. pylori* infection may increase susceptibility to severe acute respiratory syndrome coronavirus 2 (SARS-CoV-2) and other more serious forms of COVID-19, possibly due to the increased expression of angiotensin-converting enzyme II (ACE-II) receptor that permits the entry of SARS-CoV-2 and elevates the gastric pH, allowing the survival of the virus [8]. A study by Balamtekin et al. showed that abdominal pain and diarrhea in COVID-19 patients were strongly correlated with existing *H. pylori* infection in them [16]. In another study, Abdullah et al. found that *H. pylori* infection was pre-existing in patients suffering from the COVID-19 disease [17]. Furthermore, Wizenty et al. showed that there is a positive correlation between the presence of *H. pylori* and mortality resulting from COVID-19 infection [18]. Hence, dealing with the *H. pylori* problem is even more important now.

In 2017, the reality of increasing antibiotic resistance in *H. pylori* prompted the WHO to include this pathogen in a list that includes 20 pathogens that are the greatest threat to human health due to drug resistance [19]. The absence of vaccines available for diseases related to *H. pylori* prompts us to examine the status of antibiotic treatments and their limitations as well as discuss the challenges presented by the increased problem of drug resistance for this pathogen [20]. The problem of antibiotic resistance in *H. pylori* continues to rise while the efficiency of existing antibiotic treatments is reducing significantly [21]. The major challenge in preventing the rise in antibiotic resistance in *H. pylori* is the lack of proper diagnosing and treatment protocols as well as poor patient compliance. To combat this issue, it is important to continue to review the existing information while looking at the knowledge gaps that have not been addressed and finding alternative novel ways to treat the bacterium. For this study, we conducted a literature review of some articles on *H. pylori* treatment. We searched for these articles through PubMed and Google Scholar and included the most recent studies from within the past decade as well as a few older studies. We analyzed that there are no globally accepted guidelines to treat *H. pylori* infections. It is crucial to address this concern as limited literature exists on this topic. Hence, this review aims to highlight the importance of why it is challenging to establish a universally accepted protocol for antibiotic prescription. We will also delve into the critical aspects of *H. pylori* treatment and diagnosis, antibiotic resistance, the dilemma of choosing one antibiotic therapy over the other, and potential solutions to the problem. Additionally, it presents a practical chart to guide antibiotic prescription and serve the purpose of a potential foundation for developing improved universal physician guidelines. The goal is to emphasize the importance of standardized and effective treatment protocols to eradicate *H. pylori*, while also considering alternative treatment options such as plant-based therapies or optimizing drug pharmacodynamics and pharmacokinetics.

## Review

Diagnosis of *Helicobacter pylori*

The very first step in the effective treatment of *H. pylori*-related disease should be the right diagnosis of the bacterium in time [22]. Symptoms that may indicate *H. pylori* infection include epigastric pain, iron-deficiency anemia, weight loss, and dyspepsia [23]. If some of these symptoms occur, the gold standard for diagnosis is the direct histologic testing of gastric mucosal biopsies [24]. However, the efficiency of this histological approach can be affected by factors like the quality of the biopsy and the level of expertise of the pathologist performing the analysis [25]. If the treatment fails, it is recommended to do a culture and perform drug susceptibility testing [26]. According to Dore and Pes, the culture method is the current gold standard in the diagnosis of *H. pylori* as susceptibility testing can be performed accordingly [27]. However, *H. pylori* culture has proven to be cumbersome and complicated, with a significant failure rate, and requires enhanced endoscopy and diagnostic labs dedicated to this [26-28]. Molecular diagnostic tools able to identify drug resistance to pharmacological agents are increasing in importance as they circumvent the need to culture the bacteria [27].

Antibiotic-based treatment of *H. pylori* infections

Standard Triple Therapy

The classical antibiotic regimen for treating *H. pylori* infections is the standard triple therapy (STT) [29]. STT is based on the use of a proton pump inhibitor (PPI) along with one or two antibiotics, primarily clarithromycin and amoxicillin or metronidazole. STT has been the most recommended treatment method against *H. pylori* since the 90s [29]. However, its efficacy has declined from over 90% to about 70% due to increasing antibiotic resistance in *H. pylori*, especially against clarithromycin and metronidazole [29,30]. Increased clarithromycin resistance has a greater clinical impact than that of metronidazole since the efficacy of metronidazole can be increased with dose and duration of treatment [30].

Bismuth Quadruple Therapy

Due to the reducing efficacy of STT, improvisations have been made to the classical triple therapy to develop new antibiotic-based regimens for eradicating *H. pylori*. Bismuth quadruple therapy is one of these [29]. Bismuth quadruple therapy uses a PPI, plus two antibiotics: tetracycline and metronidazole, along with bismuth, for 14 days [29]. Despite being independent of clarithromycin, bismuth quadruple therapy is not exclusively considered a first-line therapy choice. Some studies from Asia, Europe, and the United States recommend this regimen as a second-line therapy choice when treatment with clarithromycin-based triple therapy has failed [24,29].

Sequential Therapy

Sequential therapy is another development in the treatment of *H. pylori*. It is also based on the STT method, except that the antibiotics are administered sequentially. For the first five days, PPI plus amoxicillin is given, while for the next five days, PPI plus clarithromycin and amoxicillin are given [29], or PPI plus clarithromycin and either metronidazole or tinidazole are administered [30]. Amoxicillin acts by disrupting the bacterial cell wall and *H. pylori* is highly susceptible to amoxicillin [29-32]. Therefore, amoxicillin is administered prior to treatment with other antibiotics to eradicate the clarithromycin- or metronidazole-resistant strains [29]. This increases the efficacy of sequential therapy [30].

Non-Bismuth Quadruple Therapy

Non-bismuth quadruple therapy is another treatment method that does not require bismuth and includes PPI, clarithromycin, amoxicillin, and metronidazole treatment for 10 days [29]. According to Goderska et al., the efficacy of this treatment method is reduced in areas of high clarithromycin resistance [29].

Levofloxacin-Based Therapy

Levofloxacin-based therapies are also being used now in regions where the resistance of *H. pylori* to quinolones is low while resistance to clarithromycin and metronidazole resistance is high, expecting eradication rates of more than 90% [29]. Some studies [29,30] recommend this option as a second-line therapy or salvage choice when treatment STT has failed, primarily due to the growing rates of levofloxacin resistance.

Although the improvised antibiotic-based treatment methods are more effective than STT in eradicating *H. pylori*, there are a few disadvantages associated with them. Table 1 compares existing antibiotic-based treatment methods used in the eradication of *H. pylori* along with their pros and cons. Since these treatment methods are based on antibiotics, growing antibiotic resistance remains a major concern [29,30].

**Table 1:** Comparison of five existing antibiotic-based therapies used in Helicobacter pylori eradication, in terms of their composition, benefits, and drawbacks. [29,30,32,33]

Treatment Method	Constituents = PPI + …	Benefits	Drawbacks
Standard Triple Therapy (STT)	Clarithromycin & Amoxicillin or Metronidazole	First-line therapy choice in areas of low clarithromycin or metronidazole resistance	Reduced efficacy due to high clarithromycin and metronidazole resistance
Bismuth Quadruple Therapy	Bismuth + Tetracycline & Metronidazole	Greater eradication rates than STT	Side effects of bismuth; non-availability of bismuth in certain regions
Sequential Therapy	Amoxicillin, Clarithromycin, & Metronidazole/Tinidazole	Greater eradication rates than STT	Reduced efficacy in areas of high clarithromycin or metronidazole resistance; allergy to amoxicillin
Non-bismuth Quadruple Therapy	Clarithromycin, Metronidazole, & Amoxicillin	No side effects from bismuth	A greater number of pills for patients; allergy to amoxicillin; reduced efficacy in areas of high clarithromycin resistance
Levofloxacin-Based Therapy	Levofloxacin & Amoxicillin/Metronidazole	Greater eradication rates than all other therapies	Prevalence of levofloxacin resistance in *H. pylori* reduces efficacy

Factors affecting *H. pylori* treatment's success

Prevalence of Antibiotic Resistance in H. pylori

The major factor in deciding the choice of treatment against *H. pylori* is antibiotic resistance [21]. *H. pylori* are now resistant to the two most widely used antibiotics in STT - clarithromycin and metronidazole, making the eradication of bacteria more difficult [29]. *H. pylori'*s resistance to antibiotics varies with region. In areas with high rates of respiratory infections and increased macrolide consumption, clarithromycin resistance is high; while in areas with high prevalence of parasitic or gynecological infections, mostly in developing countries, metronidazole resistance is high. This is because clarithromycin and metronidazole are widely used to treat the respective infections, contributing to increased antibiotic resistance rates [29].

**Figure 1:**
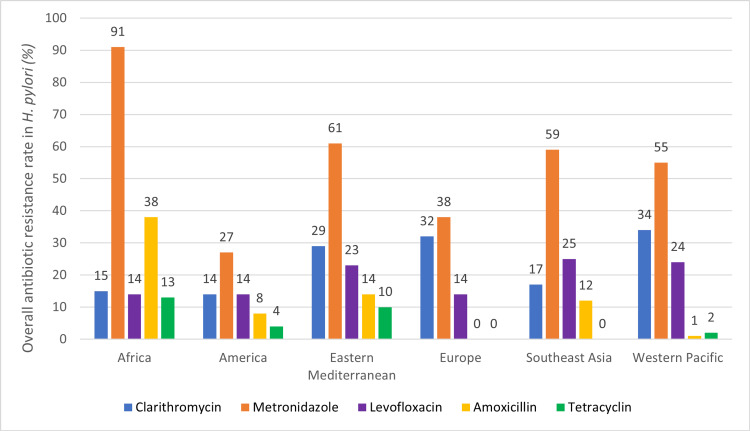
Prevalence of antibiotic resistance in Helicobacter pylori worldwide. The prevalence of antibiotic resistance in *H. pylori* for five of the major antibiotics used in *H. pylori* eradication was examined in six different WHO regions. Data presented is the mean (overall) value from pooled antibiotic resistance rates reported with a 95% confidence interval and p-value <0.05; abstracted from Katelaris et al. [28].

According to Goderska et al., the average clarithromycin resistance, as of 1997, worldwide was 17.2% and rising ever since [29]. In developing countries such as Africa, the rate of metronidazole resistance has risen to over 50%; some *H. pylori* isolates were studied to show an antibiotic resistance rate of 100%. A study of levofloxacin-based therapies depicts higher efficacy in areas of high clarithromycin or metronidazole resistance. Levofloxacin resistance increases widely in areas of high frequency of urinary tract infections since this antibiotic is primarily used to treat those infections [29]. In a study, Selgrad and Malfertheiner reported the global levofloxacin rate in *H. pylori* to be 16.2% [30]. Figure 1 shows the overall antibiotic resistance rates of major antibiotics used in the treatment of *H. pylori* infections worldwide.

Choice of Antibiotic Therapy

According to Guevara and Cogdill, antibiotic resistance is not the only factor contributing to the failure of an *H. pylori* eradication method [34]. The efficacy of a treatment regimen can also be affected by the choice of antibiotic therapy. The prescription of an antibiotic treatment chosen based on guidelines and testing of antibiotic susceptibility in cultures of *H. pylori* from infected patients can yield better eradication results [35]. Hence, the failure of STT in regions with greater than 15% clarithromycin resistance implies that the choice of antibiotic therapy affects *H. pylori* treatment outcome. Certain antibiotic-based therapies cannot be used to manage *H. pylori* infections in a region where the bacteria are resistant to that antibiotic, thus reducing the number of effective antibiotic-based treatment options available. In 2016, the Toronto Consensus guidelines proposed that clarithromycin-based triple therapy be used as a first-line intervention only in areas that do not surpass 15% of the resistance rate for this drug, or where the strain in question has been shown to be susceptible to this drug [21]. The alternative treatments would be quadruple-bismuth and non-bismuth-based approaches [21,33].

Patient Compliance with Treatment

A patient’s compliance with the treatment regimen also plays a significant role in treatment success [33]. Poor patient compliance is associated with lower treatment success [30]. Selgrad and Malfertheiner reported that patients who adhered to more than 60% of the therapy resulted in 96% treatment success while poor patient compliance reduced the efficacy of the treatment by 27% [30]. Yang et al. tested a prospective model to study the outcome of patient compliance with *H. pylori* treatment in a clinical trial [36]. The rate of *H. pylori* eradication was found to be significantly higher (>89%) in patients who complied with most of their treatment and medications than those whose compliance rates were lower (< 62.5% *H. pylori* eradication rates).

According to Yang et al., poor patient compliance to antibiotic treatment of *H. pylori* infections also contributed to increased drug resistance rates in the bacterium [36]. In a study, Axelsson reported that a patient’s adherence to an antibiotic treatment was crucial to prevent antibiotic resistance [37]. The non-adherence of patients to their treatment protocol resulted mostly when the patients were not well educated about the common side effects of the treatment and the importance of adhering to the full course of antibiotics to prevent re-infection [33,37]. Patients either missed their treatment because of side effects from the therapy or stopped taking the remaining antibiotics when they started feeling better. Any deviations from the antibiotic treatment protocol could lead to increased bacterial infection growth, requiring overuse of the antibiotic, and the bacteria developing resistance to the antimicrobial agent as a response [37]. Eventually, the antibiotic resistance developed in *H. pylori* cells contributes to treatment failure [33,38].

Choice of first-line therapy vs. second-line therapy

Existing studies reveal the higher efficacy rates of the quadruple therapy and levofloxacin-based therapy over the STT [34,39]. However, STT is still recommended as the first-line treatment choice in some places as it is challenging to standardize and establish the effectiveness of other therapies against *H. pylori* worldwide due to varying antibiotic resistance levels in different regions.

Bismuth Quadruple Therapy vs. STT

Malfertheiner et al. compared the efficacies of STT and bismuth quadruple therapy in a randomized, open-label, phase-3 trial with patients infected with *H. pylori* [39]. The study showed that the bismuth quadruple therapy had higher efficacy than that of STT with eradication rates of 80% and 55%, respectively. However, Gatta et al. found that the eradication rates of STT and bismuth quadruple therapy were similar when clarithromycin resistance rates were under 15% [40]. Therefore, in most places, quadruple therapies are being recommended as first-line treatment options where clarithromycin resistance rates are greater than 15% [34].

Sequential Therapy vs. STT

Gatta et al. further compared the efficacy of sequential therapy to that of STT in patients suffering from non-ulcer dyspepsia (NUD) and peptic ulcer disease (PUD) [40]. The results showed that sequential therapy was more effective in treating NUD with an eradication rate of 92.2% as compared to STT with an eradication rate of 73.8%. Similarly, in PUD patients, sequential therapy showed a higher eradication rate than that of STT (96% and 78.8%, respectively) [40]. Another study revealed that the efficacy of sequential therapy significantly increased when tinidazole was used instead of metronidazole during the treatment of metronidazole-resistant *H. pylori* strains [41].

Levofloxacin-based Therapies vs. STT

Levofloxacin is a broad-spectrum antibiotic that is effective against most Gram-positive and negative bacteria [34]. When levofloxacin is used to replace clarithromycin in STT or sequential therapy, the *H. pylori* eradication rates could rise to greater than 90% in contrast to STT resulting in eradication rates of less than 70% [29]. However, this is only possible in regions where levofloxacin resistance is low since it is the most widely used antibiotic in treating urinary tract infections in the USA, Europe, and Asia [29].

Levofloxacin-based Therapy vs. Other Therapies

Gisbert and Morena compared the *H. pylori* eradication rates of bismuth quadruple therapy and levofloxacin-based triple therapy (70% and 81%, respectively) [42]. Despite being more effective in eradicating *H. pylori*, levofloxacin-based regimen is not recommended as a first-line treatment option due to varied and increasing resistance rates toward this antibiotic [34] as well as the rate of adverse reactions in patients, recorded after treatment with levofloxacin-based therapies [42].

According to Vaira et al., sequential therapy is not successful in eradicating *H. pylori* in some cases [41]. Levofloxacin-based therapy is then recommended as a rescue regimen and has been studied to be successful. Liou et al. studied the levofloxacin-based regimen as both a first-line therapy choice and its possibility as a rescue therapy [43]. The results of the study showed that levofloxacin-based treatment is more effective when used as a rescue regimen after STT is not as successful as the first-line treatment. When STT was used as a rescue regimen with levofloxacin-based therapy as first-line treatment, none of the regimens were effective in treating *H. pylori* [43].

Gisbert and Morena compared the adverse effects of bismuth quadruple therapy and levofloxacin-based therapy when used as rescue regimens [42]. This study revealed that the levofloxacin-based rescue regimen showed lesser adverse effects in patients in contrast to quadruple therapy used as a rescue regimen.

Hence, the levofloxacin-based regimen is recommended as a second-line therapy choice over others while STT is still the first-line therapy choice despite lower eradication rates.

The need for an effective, standardized treatment regimen

Addressing the dilemma of choosing STT as a first-line therapy choice despite low treatment success highlights the importance of the need for a standardized treatment protocol that could be accepted worldwide. According to Qasim and O’Morain, failure of a primary treatment method complicates the eradication of *H. pylori* with second-line options [35]. Success rates of re-treatments vary and are not completely explained by the prevalence of antibiotic resistance in the bacterium [35]. Previous treatment failure or exposure to an antibiotic that is a part of the current treatment plan can significantly impact treatment success. Therefore, the choice of first-line treatment option is pivotal to the effective eradication of *H. pylori* [33].

**Figure 2:**
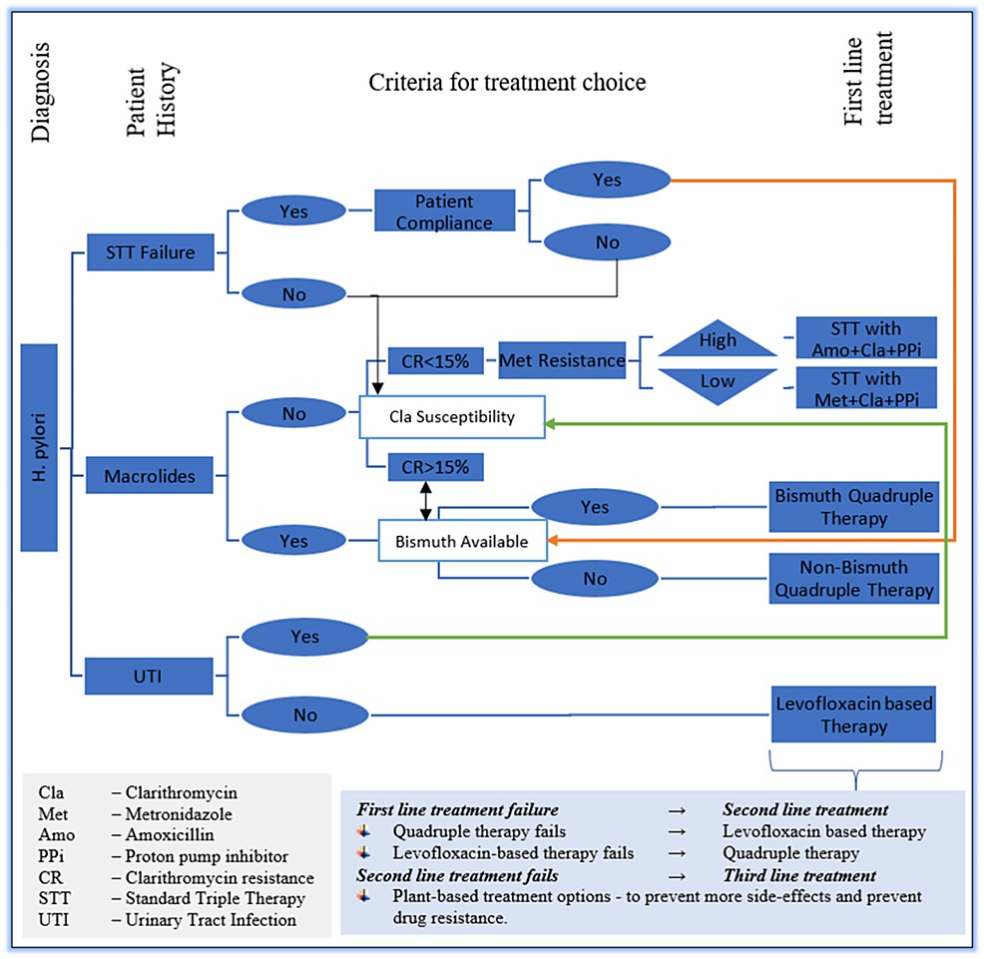
H. pylori treatment prescription protocol. 1. The first step should be the right diagnosis of *H. pylori* strain. 2. Patient history should be noted 3. Patient compliance should be considered if prior treatment has failed to eradicate *H. pylori*. At this stage, regional antibiotic susceptibility of the diagnosed *H. pylori* strain should be checked. 4. Studying factors 1-3, an antibiotic-based therapy should be chosen as a first-line option. 5. If this first-line therapy choice fails, following steps 2 through 4, a second-line therapy could be prescribed. 6. Upon failure of second-line therapy, other options such as plant-based remedies should be considered to prevent the rise in antibiotic resistance in *H. pylori* and side effects in patients. Note: This figure is original and is designed as a part of this review to illustrate a simple antibiotic therapy prescription process for *H. pylori* treatment.

According to Guevara and Cogdill, not all clinicians, gastroenterologists, or primary care physicians adhere to the expert guidelines that need to be followed when diagnosing *H. pylori* infections or prescribing the treatment needed [34]. This presents as a significant factor in reducing the efficacies of existing antibiotic-based treatment regimens while contributing to increasing antibiotic resistance rates. Enhanced measures are needed in proper diagnosing of the infection, identification of the *H. pylori* strains, assessing the antibiotic resistance of those strains, and then prescribing the proper treatment on time [29]. The Toronto Consensus guidelines emphasized the importance of considering regional antibiotic resistance rates and efficacies of existing antibiotic-based therapies before prescribing a first-line treatment regimen to patients infected with *H. pylori* [21]. To further accentuate this, we have summarized key aspects of our study and designed a simple antibiotic therapy prescription process for *H. pylori* treatment, as illustrated in Figure 2. This algorithm might lay a foundation to increase the efficacy of the remaining workable antibiotic therapies, while also reducing the time a patient needs to be treated, causing lesser adverse effects, further promoting patient compliance, and contributing to preventing a rise in antibiotic resistance rates.

Potential alternate treatment options under investigation

Some studies suggest using plants as a possible anti-*H. pylori* treatment. Neem (*Azadirachta indica*) is one such plant that has diverse medicinal properties and has been studied to be effective in treating *H. pylori* infections, both *in vitro* [44,45] and *in vivo* [46]. Garlic is another plant-based option that has been shown to provide bacteriostatic effects against *H. pylori *in infected patients and has the potential to be an effective bactericidal agent to eradicate *H. pylori *[47]. Plant-based treatment methods might also help in combating the growing antibiotic resistance in *H. pylori*. When both first- and second-line treatment regimens have failed against *H. pylori*, alternate options like plants should be preferred to treat the infections to lower the adverse effects from continuous use of antibiotic-based therapies and prevent further growth in antibiotic resistance rates.

Novel treatment regimens against *H. pylori* should also consider the pharmacodynamics and pharmacokinetics of the drugs. Pairing drugs with similar kinetics might lead to optimal bioavailability and enhanced antibacterial effect [48].

The study of pharmacodynamics and pharmacokinetics of the active anti-*H. pylori* ingredients in Neem plant and garlic may reveal the possibility of including these bactericidal agents in antibiotic-based regimens. This might lead to effective eradication of the bacterium without contributing to antibiotic resistance or side effects and could be a breakthrough in *H. pylori* treatment while promoting patient compliance.

## Conclusions

Eradication of *H. pylori* infections remains a global health challenge, especially in the context of its association with gastric cancer as well as the severity of COVID-19 disease. The rising rates of antibiotic resistance highlight the urgency of effective treatment strategies. The lack of an effective treatment option against *H. pylori* presents a significant barrier in combating the problem, while the incidence of infections and the burden of disease associated with the pathogen continue to grow. The goal should be to eradicate the bacterium with the first treatment and avoid subsequent therapies to prevent further increases in the rates of antibiotic resistance in *H. pylori*. Implementing a standardized prescription protocol could be a potential starting point for the development of universally accepted physician guidelines to treat *H. pylori*. Establishing these guidelines is imperative to enhance *H. pylori* infection management globally while promoting patient compliance with the treatment. Furthermore, more research on the effectiveness of the Neem plant, other herbal remedies, and the role of pharmacodynamics and pharmacokinetics in treating *H. pylori* infections is needed. If a plant-based treatment results in eradication of the bacterium without serious side effects, it will be a promising avenue especially when conventional antibiotic treatment regimens fail. This alternative might also address the concerns of rising antibiotic resistance rates in *H. pylori*.

Overall, this review sheds light on the need for an effective globally accepted treatment strategy which is a possibility if we investigate the concerns regarding *H. pylori* treatment, diagnosis, antibiotic resistance, and alternative novel approaches for treatment, altogether. Collaborative efforts among researchers, physicians, clinicians, and government health policymakers will be crucial in addressing these concerns and combating the *H. pylori* problem.
